# Marriage and childbearing in patients with epilepsy in Turkey

**DOI:** 10.3389/fneur.2024.1304076

**Published:** 2024-03-21

**Authors:** Firdevs Ezgi Uçan Tokuç, Fatma Genç, Eylem Özaydın Göksu, Abidin Erdal, Yasemin Biçer Gömceli

**Affiliations:** ^1^Department of Neurology, Antalya Training and Research Hospital, Antalya, Türkiye; ^2^Department of Neurology, Health Ministry of Türkiye Republic, Ankara Bilkent City Hospital, Ankara, Türkiye; ^3^Department of Neurology, Memorial Antalya Hospital, Antalya, Türkiye

**Keywords:** patient with epilepsy, stigma, marriage, childbearing, social support

## Abstract

**Introduction:**

For epilepsy, a common neurological disorder, brings psychosocial challenges like stigma, employment difficulties, and barriers to marriage and childbearing. Stigma often stems from misconceptions and societal beliefs, particularly in less developed regions like Turkey. However, research on the marital and childbearing experiences of epilepsy patients in such settings is limited. We aimed to research the marriage and childbearing behaviors of men and women with epilepsy.

**Methods:**

We conducted a cross-sectional study involving 215 adult epilepsy patients at Antalya Training and Research Hospital between 2019 and 2022. Patients were asked questions about marriage and having children on prepared questionnaires.

**Result:**

The gender distribution of the 215 patients included in the study was revealed to be 62.3% (134) females and 37.7% (81) males. 71.6% of patients were married, and 12.7% had no children. 33.3% of these patients stated that they did not desire children because of the disease. A statistically significant correlation was observed between the duration of the disease and being unmarried. A significant correlation was observed between age at disease onset and number of children.

**Conclusion:**

Our study revealed the effects of individuals with epilepsy on marriage and childbearing, and as we know, it is the first study conducted in Turkey on childbearing attitudes in individuals with epilepsy. Despite medical and social developments, epilepsy is still one of the most stigmatized diseases, and the disease has considerable negative effects on marriage and fertility. Our study supported the findings of a small number of previous similar studies on this subject and additionally showed that the likelihood of having children decreased in patients using multiple ASM, and on the other hand, it showed that marriage positively affected patients in terms of social support.

## 1 Introduction

Epilepsy is one of the most common neurological diseases, and in addition to the problems caused by seizures, these patients also deal with psychosocial problems such as finding a job, stigma, marriage, and having children (1, 2). Dealing with such psychosocial issues may sometimes be more challenging than managing seizures (1, 2). In recent years, many studies have been conducted on the stigmas experienced by patients with epilepsy and have shown that the stigmas experienced are related to personal variables such as age, education level, and economic status, as well as the type and frequency of seizures (1–4).

The most prominent causes of stigma are false beliefs and social attitudes. Particularly in underdeveloped countries, there are thoughts that epilepsy is a mental disease or a punishment for the sins of individuals (5). In a community-based study conducted by Nicholes et al. 19% of the population considered epilepsy a psychological disease, 5.2% thought that epilepsy was caused by a supernatural power, 13% refused to work with individuals with epilepsy, and approximately half (45.4%) refused to marry these patients (6). In another study conducted in the USA, 37% of the individuals who participated in the study stated that epilepsy was a mental illness, 20% stated that it was an infectious dis-ease, and 13% stated that it emerged as a punishment for sin (7). In a study including 346 teachers in Turkey, 40% of the teachers reported that they did not want to marry individuals with epilepsy, and 73% reported that they would not allow their children to marry individuals with epilepsy (8). This kind of negative thinking and attitude toward epilepsy causes patients to isolate themselves from society, conceal their disease, and avoid marriage and having children (2, 4). Studies on marital behavior and childbearing in patients with epilepsy are mostly conducted in developed countries, and these studies have shown that individuals with epilepsy are less likely to marry compared to the general population and that especially being diagnosed with epilepsy at an early age reduces the possibility of marriage (8, 9). It is also noteworthy that individuals with epilepsy not only have lower rates of marriage but also higher rates of divorce (10). Moreover, small number of studies conducted in developed countries have shown that both women and men have a decreased tendency to have children (11). However, data in this regard is quite limited in developing countries. In light of these data, we aimed to research the marriage and childbearing behaviors of men and women with epilepsy. In addition, this is the first study on childbearing behaviors of patients with epilepsy in Turkey.

## 2 Materials and methods

### 2.1 Patients and procedures

This descriptive and cross-sectional study has been conducted in the epilepsy outpatient clinic of Antalya Training and Research Hospital, which is a 3rd level center in Turkey and where approximately 4000 epilepsy patients are followed up. Adult patients who came to routine epilepsy outpatient clinic follow-up between 2019 and 2022 were enrolled. A face-to-face interview with a semi-structured questionnaire was conducted in the epilepsy outpatient clinic by a neurologist. Epilepsy was classified according to the new 2017 International League Against Epilepsy (ILAE) classification.

The inclusion criteria were as follows: (1) over the age of 18 men and women, (2) at least 1-year diagnosis of epilepsy, (3) use at least 1 anti-seizure medication (ASM). Patients who were not receiving ASM, had an intellectual disability, or had psychiatric diseases affecting quality of life were excluded from the study.

Prior to the study, the approval of the Ethics Committee of Antalya Training and Research Hospital was obtained, and an informed consent form was completed by all patients.

### 2.2 Data collection

Patients were asked questions on prepared surveys. The questions included in the survey were divided into 3 sections. The first section included demographic variables (age, gender, education level, employment status) and epilepsy profile (period of disease, age of disease onset, number and names of ASM used, classification of seizures, epilepsy remission status). The second section consisted of questions about the marital status of the patients and the effects of their disease on their marriages. In the third section of the survey, the status of having children and the stigmas experienced due to the disease were questioned (Additional file 1).

### 2.3 Data analysis

The data were analyzed with IBM SPSS V23. Compliance with the normal distribution was examined by the Shapiro–Wilk and Kolmogorov–Smirnov tests. Categorical data were analyzed with Yates Correction, Fisher’s Exact Test, and Pearson’s Chi-Square Test, and the Bonferroni-Adjusted Z Test was utilized for multiple comparisons. The Mann–Whitney U Test was utilized for the comparison of variables that complied with normal distribution in paired groups, and the Independent Samples *t* Test was utilized for those that did not comply with normal distribution. The One-way ANOVA was performed for the comparison of data complying with the normal distribution in groups of three or more, and multiple comparisons were performed with the Tamhane Test. The Kruskal-Wallis Test was applied for the comparison of data that did not comply with normal distribution in groups of three or more, and multiple comparisons were made with Dunn’s Test. The results of the analysis were presented as frequency (percentage) for categorical variables, mean ± standard deviation, and median (minimum-maximum) for quantitative variables. The Significance level was taken as *p* < 0.050.

## 3 Results

The gender distribution of the 215 patients included in the study was revealed to be 62.3% (134) females and 37.7% (81) males. The mean age of the patients was found to be 35.31 ± 10.25 years. When the distribution of education levels was examined, the highest proportion was high school graduates with 34.4%. The demographic data of the patients and their categorical distribution as per the survey questions are summarized in Table 1.

**Table 1:** Frequency distributions of variables and descriptive statistics.

	Frequency /Mean ± sd	Percentage/ median (min–max)
Gender		
Female	134	62.3
Male	81	37.7
Age	35.31 ± 10.25	34 (18–67)
Educational level		
Illiterate	2	0.9
Primary education	107	49.8
Secondary education	74	34.4
University	32	14.9
Duration of disease	16.42 ± 10.91	15 (1–49)
Age of disease onset	19.13 ± 10.97	17 (0–55)
Number of anti-seizure medications used	1.61 ± 1.06	1 (0–9)
Did you have epilepsy before you got married?		
No	55	32.9
Yes	112	67.1
Classification of seizures		
Generalized epilepsy	40	18.6
Focal epilepsy	151	70.2
Unclassifiable	24	11.2
Epilepsy remission status		
Seizure-free	156	72.9
Seizures continued	54	25.2
Undetermined	4	1.9
Having a profession and employment		
Has a job	87	40.5
Have problems at job	7	3.3
Have difficulty finding a job	26	12.1
Unable to work due to disease	38	17.7
Home-not employed	57	26.5
Married?		
Single	61	28.4
Married	154	71.6
Unmarried patients-why aren’t you married?		
Due to epilepsy	12	21.4
Other	44	78.6
Do you ever feel labeled, stigmatized?		
No	141	66.2
Yes	72	33.8
Do you feel you are different from other people?		
No	145	68.1
Yes	68	31.9
Do you receive adequate social support from your family?		
No	29	13.6
Yes	184	86.4
Do you have a boyfriend/girlfriend?		
No	47	77
Yes	14	23
How old did you get married?	23.17 ± 4.34	22 (15–35)
Number of marriages you are in?		
1	158	94.6
2	9	5.4
Does your spouse have a health problem?		
No	142	89.3
Yes	17	10.7
Are you happy in your marriage?		
No	11	7.1
Yes	143	92.9
Does your disease negatively affect your marriage?		
No	129	82.2
Yes	28	17.8
Did you disclose your disease to your spouse or his/her family before you got married?		
No	35	31
Yes	78	69
Did your spouse/his-her family have any prejudice against your disease?		
No	131	84
Yes	25	16
Did Your marriage affect your regular check-ups and regular medication?		
No	148	93.7
Yes	10	6.3
Did your partner understand your disease?		
No	9	5.7
Yes	149	94.3
Do you have sexual problems with your partner due to your disease?		
No	120	75.9
Yes	38	24.1
Was your disease a factor in your divorce?		
No	8	38.1
Yes	13	61.9
Did hiding your disease before marriage have negative effects?		
No	15	88.2
Yes	2	11.8
Who wanted to get divorced?		
Patient	13	61.9
His/her Spouse	2	9.5
Both	6	28.6
Did you have a second marriage?		
No	12	57.1
Yes	9	42.9
Do you have children, and if yes, how many?		
No	21	12.7
1	63	38
2	57	34.3
3	22	13.3
4	3	1.8
Did you not desire it because of your disease?		
Due to disease	7	33.3
Did Not Desire	10	47.6
Due to Spouse	4	19

When the patients were compared according to gender, the correlation between having a profession/employment status according to gender was found to be statistically significant. Males were more likely to work without any problems in their jobs than females, whereas females had a higher rate of being at home and not employed (*p* < 0.001).The correlation between the rate of difficulty in taking care of a child due to disease according to gender was found to be statistically significant (*p* = 0.016) and the rate of those who had difficulty in taking care of a child due to disease was 31.5% among females and 11.8% among males. No significant correlation was found between gender and other variables (*p* > 0.050).

71.4% of the patients were married, and 31% of these patients concealed the disease from their spouses. The correlation between those who received adequate social support from their families and being married was found to be statistically significant (*p* = 0.004). This rate was higher in married patients. No significant correlation was found between being married and other variables (*p* > 0.050).

A statistically significant difference was found between the median values of the duration of disease in married and single patients (*p* = 0.027). The median duration of disease for single patients was 11, while the median duration of disease for married patients was 16. A statistically significant difference was found between the mean ages of the participants according to their marital status (*p* < 0.001). The mean age of single patients was 30.72, whereas the mean age of married patients was 37.12. No significant difference was found between being married and other variables (*p* > 0.050) (Tables 2, 3).

**Table 2:** Comparison of categorical data according to being married.

	Married?		Test statistics	*p*
	Single	Married		
Gender				
Female	32 (52.5)	102 (66.2)	2.968*	0.085
Male	29 (47.5)	52 (33.8)		
Educational level				
Illiterate	1 (1.6)	1 (0.6)	8.318**	**0.040**
Primary education	21 (34.4)^a^	86 (55.8)^b^		
Secondary education	28 (45.9)^a^	46 (29.9)^b^		
University	11 (18)	21 (13.6)		
Classification of seizures				
Generalized epilepsy	12 (19.7)	28 (18.2)	3.352**	0.187
Focal epilepsy	46 (75.4)	105 (68.2)		
Unclassifiable	3 (4.9)	21 (13.6)		
Epilepsy remission status				
Without seizure	42 (68.9)	114 (74.5)	2.969**	0.227
Seizure continued	19 (31.1)	35 (22.9)		
Undetermined	0 (0)	4 (2.6)		
Having a profession and employment				
Has a job	27 (44.3)	60 (39)	21.652**	**<0.001**
Have problems at job	0 (0)	7 (4.5)		
Have difficulty finding a job	14 (23)^a^	12 (7.8)^b^		
Unable to work	14 (23)	24 (15.6)		
Home-not employed	6 (9.8)^a^	51 (33.1)^b^		
Do you ever feel labeled, stigmatized?				
No	37 (62.7)	104 (67.5)	0.254**	0.614
Yes	22 (37.3)	50 (32.5)		
Do you feel you are different from other people?				
No	40 (67.8)	105 (68.2)	0.000**	1.000
Yes	19 (32.2)	49 (31.8)		
Do you receive adequate social support from your family?				
No	15 (25.4)	14 (9.1)	8.336**	**0.004**
Yes	44 (74.6)	140 (90.9)		

*Perason’s Chi-Square Test; **Yates Correction; a-b: There is no difference between groups with the same letter. Bold variables disclosing statistical significance (*p* < 0.05).

**Table 3:** Comparison of quantitative data according to being married.

	Married?				Test statistics	*p*
	Single		Married			
	Mean ± sd	Median (min–max)	Mean ± sd	Median (min–max)		
Age	30.72 ± 8.93	29 (18–54)	37.12 ± 10.2	37 (20–67)	-4.292*	**<0.001**
Duration of disease	13.93 ± 10.47	11 (1–49)	17.4 ± 10.95	16 (1–46)	5.605.5**	**0.027**
Age of disease onset	16.89 ± 8.63	16 (0–46)	20.03 ± 11.67	18 (0–55)	5383**	0.095
Number of ASM used	1.64 ± 1	1 (1–5)	1.6 ± 1.08	1 (0–9)	4490**	0.560

*Independent samples *t* Test; **Mann Whitney U Test, ASM, Anti-seizure Medication. Bold variables disclosing statistical significance (*p* < 0.05).

12.7% of the patients did not have children, and 33.3% of these patients stated that they did not desire children because of the disease. A statistically significant difference was found between the mean age at onset of the disease according to the number of children the patients had (*p* = 0.025). Patients with epilepsy at an early age were observed to have fewer children. The median age at onset of the disease was 13 years for those who did not want children due to the disease and 19.5 years for those who did not want children regardless of the disease which was statistically significant (*p* = 0.033). Age at onset of disease did not differ according to other variables (*p* > 0.050).

When categorical data were compared with age at onset of disease, period of disease, seizure classification, number of ASMs used, and epilepsy remission status, a statistically significant difference was found between the median age at onset of disease according to feeling that they were different from other people (*p* = 0.022). The median age of onset was 16 years for those who felt differently and 18 years for those who did not. A statistically significant difference was found between the median values of the period of disease and feeling that they were different from other people (*p* = 0.015). The period of disease was observed to be longer for those who felt that they were different from other people.

A statistically significant difference was found between the median values of the duration of disease according to being married (*p* = 0.027). While the median value of the duration of disease was 11 for single patients, this value was calculated as 16 for married ones. According to other variables, the period of disease does not display a statistically significant difference (*p* > 0.050).

No statistically significant correlation was observed between seizure classification and the variables (*p* > 0.050).

A statistically significant difference was found between the median value of the ASMs used and having a profession and employment status (*p* = 0.005). The number of ASMs used was observed to be higher for patients who were unable to work or who had problems at work. A statistically significant difference was found between the median values of the number of ASMs used according to the difficulty in taking care of their child due to the disease (*p* = 0.010). The median value of the number of ASMs used by those who had difficulty taking care of their children due to disease was observed to be higher.

According to epilepsy remission status, the correlation between the disease and having sexual problems with the spouse was found to be statistically significantly higher among those whose seizures continued (*p* = 0.009). No statistically significant correlation was found between epilepsy remission status and other variables (*p* > 0.050).

The correlation between having epilepsy before marriage and the number of children was found to be statistically significant (*p* < 0.001). Among those who had epilepsy before marriage, the proportion of those having 1 child was 56.5%, that of 2 children was 33.7%, that of 3 children was 8.7%, and that of 4 children was 1.1%. No statistically significant correlation was found between having epilepsy before marriage and other variables (*p* > 0.050).

Risk factors for not having children were analyzed by binary logistic regression analysis. As a result of the univariate analysis, it was concluded that the risk of not having children was 6 times higher for university graduates compared to primary education graduates (*p* = 0.002). When analyzed in terms of epilepsy remission status, the risk of not having children was observed to be 7.714 times higher in those in the undetermined category compared to those without seizures (*p* = 0.049). The risk of not having children increases 1.057 (1/0.946) times as the age at onset of the disease decreases (*p* = 0.036). Other variables were not significant in the univariate analysis. In the multiple analysis, the risk of not having children was 4.039 times higher for university graduates compared to primary school graduates, while the risk of not having children increased 1.072 times as the age at onset of the disease decreased (*p* values 0.044 and 0.042, respectively) (Table 4). There was no statistically significant difference between those who did not want to have children and gender (*p* = 0.08).

**Table 4:** Risk factors that affect not having children.

	Having children		Univariate		Multiple	
	Yes	No	OR (%95 CI)	*p*	OR (%95 CI)	*p*
Educational level						
Primary education	84 (92.3)	7 (7.7)	Reference			
Secondary education	45 (88.2)	6 (11.8)	4.600 (0.507−5.048)	0.423	1.895 (0.543−6.616)	0.317
University	16 (66.7)	8 (33.3)	6.000 (1.906−18.886)	**0.002**	4.039 (1.036−15.743)	**0.044**
Gender						
Female	92 (83.6)	18 (16.4)	3.457 (0.973−12.285)	0.055		
Male	53 (94.6)	3 (5.4)	Reference			
Epilepsy remission status						
Seizure-free	108 (88.5)	14 (11.5)	Reference			
Seizures continued	34 (87.2)	5 (12.8)	1.134 (0.381−3.379)	0.821	2.535 (0.572−11.236)	0.221
Undetermined	2 (50)	2 (50)	7.714 (1.006−59.180)	**0.049**	10.109 (0.789−129.579)	0.075
Profession						
Has a job	53 (82.8)	11 (17.2)	0.524 (0.209-1.315)	0.169		
Has no job	92 (90.2)	10 (9.8)	Reference			
Age of disease onset	20.7 ± 12.1	15 ± 5.9	0.946 (0.899−0.996)	**0.036**	0.933 (0.873−0.998)	**0.042**
Number of ASM used	1.6 ± 1.1	1.3 ± 0.5	0.604 (0.300−1.219)	0.159	0.458 (0.156−1.35)	0.157

ASM, Anti-seizure Medication. Bold variables disclosing statistical significance (*p* < 0.05).

## 4 Discussion

Our study revealed the effects of individuals with epilepsy on marriage and childbearing, and as we know, it is the first study conducted in Turkey on childbearing attitudes in individuals with epilepsy. A total of 28.4% of our patients were single, and 12% of these patients reported that they did not marry due to epilepsy. In a previous study, similar to our study, it was found that the rate of marriage in epilepsy patients was only 80%, and they found that three out of four of the single patients did not marry due to epilepsy. The reason for this was attributed to the interaction between both the externalized (social discrimination they experience) and internalized stigmas of epilepsy (negative experiences they may have in marriage negotiations due to public attitudes) (10). No statistical difference was observed between marriage and epilepsy remission status, as well as seizure classification. In many earlier studies, it was reported that having active seizures or clinical features related to seizures did not lead to any negative attitude toward current marital status (11, 12).

In addition, studies have found a correlation between the duration of disease and marital status, and it was observed that the period of epilepsy was longer and the age at onset of epilepsy was earlier in single patients (1, 13, 14). The data in our study were consistent with the opposite. Married patients had a longer disease period. The reason for this is the fact that a significant difference was found between the mean ages of the participants according to being married in the patient population participating in our study, and the mean age of the married participants was higher than the single participants (*p* < 0.001). Therefore, we consider that the reason for these findings that are inconsistent with the literature is related to this situation.

In our study, the proportion of married patients receiving adequate social support was notably higher. It is well known that marriage is the most significant factor contributing to social support. However, studies have shown that this is not the case with epilepsy. Patients with epilepsy do not receive enough support from their partners and therefore tend to conceal their disease. This promising detail observed in our study may be an indication that awareness has increased in the young population, and consequently, patients receive support from their spouses rather than their families. Further studies in this regard may strengthen our hypothesis.

Although there is an inverse relationship between higher education level and having children, the factors affecting this are quite complex and chronic diseases such as epilepsy may affect this situation even more (15). In our study, the risk of having children decreased with increasing educational level in epilepsy patients. There may be several reasons for this. As the level of education increases, more information about the disease and medications can be obtained through tools such as the internet and books. Therefore, women may have concerns about the side effects of medications on babies during pregnancy. In addition, the fact that epilepsy patients think that they may have difficulty taking care of their babies may also affect this decision (16). Also, several studies have found that female epilepsy patients do not want to have children more than male epilepsy patients (14, 16). However, no gender difference was found in our study. This may be due to the fact that there are relatively few patients who do not have children. When we analyzed the factors affecting having children, we found that as the number of ASMs increased, the rate of having children decreased and also it was established that patients in our population had fewer children than the national population There may be several reasons for this. One of the biggest hesitations is the fear that persistent medication-related damage may develop in the children of the patients, and another important factor is that patients who take more than one ASM are considered to have refractory epilepsy, the concern of not being able to have a job due to the disease and having difficulty taking care of their children (1).

In addition, individuals with epilepsy experience extreme stigmatization. In our study, 33.8% of our patients stated that they felt labeled or stigmatized, while 31% stated that they felt that they were different from other patients. In an earlier study conducted by Yeni et al. in Turkey in 2016, it was reported that 34% of individuals with epilepsy experienced stigma, and they stated that this rate was similar to other previous studies. Moreover, they argued that the rates of stigmatization were similar to the studies conducted in Europe 16 years ago, and although there have been medical advances in epilepsy in the last 16 years, no progress has been achieved to reduce stigmatization (4). Whereas 39.8% stigma was observed in a study conducted in China in 2022, this rate increased to 62.4% in a study conducted in eastern Turkey (17, 18). These patients, for whom stigma is significantly severe, are afraid of inheriting their disease through genetic transmission to their descendants. This is one of the reservations about having a child (1).

Also, sexual dysfunctions related to both epilepsy and medications are frequently encountered in individuals with epilepsy (19–21). In our study, patients with continued seizures reported that they had statistically significant sexual problems with their spouses. It should be noted that this may be one of the underlying reasons for low childbearing rates.

Furthermore, the rate of those who had difficulty taking care of their children was 24.3% in our study, and this rate was statistically significantly higher in women and in those taking multiple ASM. Patients most frequently reported difficulties when it came to care, and 18.2% of these patients did not receive any assistance. Spousal support for childcare is available only for 27.3% of them. We believe that this has a negative impact on their thoughts about having children again.

There are various limitations in our study. First, we did not use a stigma measurement survey in our study. Only patients’ feelings of being labeled/stigmatized and feeling that they were different from other people were questioned. If stigma tests had been applied, more accurate data could have been obtained in terms of labeling and internal stigmatization. Another limitation of our study was that there were no control groups either healthy or with any other chronic illness. In addition, the low number of male patients included in our study may also be a cause of bias.

## 5 Conclusion

In conclusion, despite medical and social developments, epilepsy is still one of the most stigmatized diseases, and the disease has considerable negative effects on marriage and fertility. Our study supported the findings of a small number of previous similar studies on this subject and additionally showed that the likelihood of having children decreased in patients using multiple ASM, and on the other hand, it showed that marriage positively affected patients in terms of social support. Greater collective efforts are needed to create a better environment in which people with epilepsy can be well understood and supported.

## Data availability statement

The original contributions presented in the study are included in the article/Supplementary material, further inquiries can be directed to the corresponding author.

## Ethics statement

The studies involving humans were approved by Institutional Review Board (or Ethics Committee) of University of Antalya Training and Research Hospital. The studies were conducted in accordance with the local legislation and institutional requirements. The participants provided their written informed consent to participate in this study.

## Author contributions

FEUT: Conceptualization, Investigation, Methodology, Writing – original draft, Writing – review & editing. FG: Conceptualization, Investigation, Methodology, Writing – review & editing. EÖG: Resources, Writing – original draft. AE: Writing – review & editing. YBG: Conceptualization, Investigation, Methodology, Writing – original draft.
