# Supplementary material for: Marriage and childbearing in patients with epilepsy in Turkey
Source: Front Neurol. 2024 Mar 21;15:1304076. doi: 10.3389/fneur.2024.1304076 (PMC10996395; doi:10.3389/fneur.2024.1304076)
Supplement: Supplementary file 1 [file Data_Sheet_1.docx]

Marriage and Childbearing in Patients with Epilepsy in Turkey

**DEMOGRAPHİC DATA AND EPİLEPSY PROFİLE**

Name-Surname:

File No:

Age:

Gender:

Education level:

Illıterate:

Primary Education:

Secondary Education:

University:

Employment status:

Has a Job:

Have Problems at Job:

Have Difficulty Finding a Job:

Unable to work due to disease:

Home-Not Employed:

Duration of disease:

Age at Disease Onset:

Number and names of ASMs used:

Classification of seizures:

Generalized Epilepsy:

Focal Epilepsy:

Unclassifiable:

Epilepsy remission status:

Seizure- free:

Seizure continued:

Undetermined:

Did you have epilepsy before marriage?

MARTİAL STATUS AND THE EFFECTS OF THEİR DİSEASE ON THEİR MARRİAGES

Martial Status:

Single:

Married:

If Single: Why aren’t you married?:

Due to epilepsy:

Other:

Number of marriage you are in? :

Do you have a boyfriend/girlfriend?

Yes:

No:

How old are you get married? :

Are You Happy in Your Marriage?

Yes:

No:

Does Your Spouse Have a Health Problem?

Yes:

No:

Does Your Disease Negatively Affect Your Marriage?

Yes:

No:

Did you disclose your disease to your spouse or his/her family before you got married?

Yes:

No:

Did your spouse/his-her family have any prejudice against your disease?

Yes:

No:

Did Your Marriage Affect Your Regular Check-ups and Regular Medication?

Yes:

No:

Did your partner understand your disease?

Yes:

No:

Do you have sexual problems with your partner due to your disease?

Yes:

No:

Did Hiding Your Disease Before Marriage Have Negative Effects?

Yes:

No:

Are you divorced?

Yes:

No:

Who wanted to get divorced?

Patient:

His/Her Spouse:

Both:

Was your disease a factor in your divorce?

Yes:

No:

Did You Have a Second Marriage?

Yes:

No:

**THE STİGMAS EXPERİENCED DUE TO THE DİSEASE AND THE STATUS OF HAVİNG CHİLDREN AND**

Do you feel labeled or stigmatized?

Yes:

No:

Do You Feel You Are Different from Other People?

Yes:

No:

Do you receive adequate social support from your family?

No

Yes

Do you have children, and if yes, how many? :

Did you not desire it because of your disease?

Due to disease:

Did Not Desire:

Due to Spouse:
